# Detection of *Campylobacter jejuni* diversity by clustered regularly interspaced short palindromic repeats (CRISPR) from an animal farm

**DOI:** 10.1002/vms3.622

**Published:** 2021-09-12

**Authors:** Hung‐Yueh Yeh, Amal Awad, Michael J. Rothrock

**Affiliations:** ^1^ Poultry Microbiological Safety and Processing Research Unit, U.S. National Poultry Research Center, Agricultural Research Service United States Department of Agriculture Athens Georgia USA; ^2^ Bacteriology, Mycology and Immunology Department, Faculty of Veterinary Medicine Mansoura University Mansoura Egypt; ^3^ Egg Safety and Quality Research Unit, U.S. National Poultry Research Center, Agricultural Research Service United States Department of Agriculture Athens Georgia USA

**Keywords:** *Campylobacter jejuni*, clustered regularly interspaced short palindromic repeats, CRISPR

## Abstract

**Background:**

*Campylobacter jejuni* is the leading bacterial pathogen that causes foodborne illness worldwide. Because of genetic diversity and sophisticated growth requirements of *C. jejuni*, several genotyping methods have been investigated to classify this bacterium during the outbreaks. One of such method is to use clustered regularly interspaced short palindromic repeats (CRISPR).

**Objectives:**

The goal of this study was to explore the diversity of *C. jejuni* isolates with CRISPR from an animal farm.

**Methods:**

Seventy‐seven *C. jejuni* isolates from an animal farm were used in this study. The day‐old broilers were reared with other poultry and farm animals, including layer hens, guinea hens, dairy goats and sheep. A small swine herd was also present on an adjacent, but separate plot of land. Isolation and identification of *C. jejuni* were performed according to the standard procedures. The CRISPR type 1 was PCR amplified from genomic DNA, and the amplicons were sequenced by the Sanger dideoxy method. The direct repeats (DRs) and spacers of the CRISPR sequences were identified using the CRISPRFinder.

**Results:**

The CRISPR sequences were detected in all 77 isolates. One type of DRs was identified in these 77 isolates. The lengths of the CRISPR locus ranged from 100 to 560 nucleotides, whereas the number of spacers ranged from one to eight. The distributions of the numbers of CRISPR spacers from different sources seemed to be random. Overall, 17 out of 77 (22%) *C. jejuni* isolates had two and five spacers, whereas 14 out of 77 (18%) isolates had three spaces in their genomes. By further analysis of spacer sequences, a total of 266 spacer sequences were identified in 77 *C. jejuni* isolates. By comparison with known published spacer sequences, we observed that 49 sequences were unique in this study. The CRISPR sequence combination of Nos. 16, 19, 48 and 57 was found among a total of 15 *C. jejuni* isolates containing various multi‐locus sequence typing (MLST) types (ST‐50, ST‐607, ST‐2231 and ST‐5602). No. 57 spacer sequence was unique from this study, whereas the other three (Nos. 16, 19 and 48) sequences were found in previous reports. Combination of Nos. 5, 9, 15, 30 and 45 was associated with ST‐353. To compare the CRISPR genotyping with other methods, the MLST was selected due to its high discriminatory power to differentiate isolates. Based on calculation of the Simpson's index of diversity, a combination of both methods had higher Simpson's index value than those for CRISPR or MLST, respectively.

**Conclusions:**

Our results suggest that the MLST from *C. jejuni* isolates can be discriminated based on the CRISPR unique spacer sequences and the numbers of spacers. In the future, investigation on the CRISPR resolution for *C. jejuni* identification in outbreaks is needed. A database that integrates both MLST sequences and CRISPR sequences and is searchable is greatly in demand for tracking outbreaks and evolution of this bacterium.

## Graphical abstract

Fig. 1 Distribution of the numbers of CRISPR spacers in *Campylobacter jejuni* isolates from an animal farm in 2016. The x‐axis indicates the numbers of CRISPR spacers. The y‐axis represents the numbers of *Campylobacter jejuni* isolates.

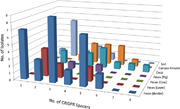

## 1 INTRODUCTION

Clustered regularly interspaced short palindromic repeats (CRISPR) was first described by Ishino et al. (1987) that the highly unusual repetitive sequences of 29 nucleotides (as direct repeats [DRs]) were regularly spaced with 32 nucleotides (as spacers) during their study on the *Escherichia coli iap* gene. Since then, this similar pattern has been found in genomes of many archaea and prokaryotes (Ishino et al., 2018). The striking characteristics of the CRISPR pattern are as follows: (1) DRs interspaced with various numbers of unique, non‐repetitive sequences (so‐called spacers), (2) a leader sequence at the one side of the locus acting as a promoter and (3) various numbers of the *cas* family genes (**C**RISPR‐**a**ssociated **g**enes) (Grissa et al., 2009; Ishino et al., 2018). The CRISPR–Cas systems in prokaryotes and archaea play important roles in defence of infecting bacteriophages and plasmids (Barrangou and Horvath, 2009; de Cardenas et al., 2015; Louwen et al., 2014). Also, these systems may act as virulence factors during bacterial pathogenesis (Ahmed et al., 2018; Hille et al., 2018). Recently, the CRISPR has been used for genotyping foodborne pathogen *Salmonella* to track outbreaks and evolution (e.g., Shariat and Dudley, 2014; Cox et al., 2019).

*Campylobacter jejuni*, a Gram‐negative bacterium (Ryan et al., 2004; Ursing et al., 1994), is the leading foodborne pathogen worldwide (Kirk et al., 2015; Tack et al., 2019). It is estimated that this bacterium causes about 1.3 million cases of human campylobacteriosis in the U.S. annually (Crim et al., 2015; Scallan et al., 2011). The reservoirs of *C. jejuni* are found in guts of many animals where this bacterium is regarded as a member of gut microbiomes (Hermans et al., 2012; European Food Safety Authority, 2010). Therefore, control of this bacterium is extremely difficult (Lin, 2009; Sahin et al., 2015). In addition, because of the genetic diversity and sophisticated growth condition of *C. jejuni*, detection and identification of this bacterium with classic culture methods are problematic (On et al., 2003). Many genotyping methods for this bacterium have been investigated to solve these problems. One such method is CRISPR (de Cárdenas et al., 2015; Kovanen et al., 2014; Louwen et al., 2013).

In this short communication, we applied the CRISPR typing to explore the diversity of *C. jejuni* isolates from an animal farm in 2016.

## 2 MATERIALS AND METHODS

### 2.1 Bacterial cultures and genomic DNA isolation

Seventy‐seven *C. jejuni* isolates from 2016 and an animal farm were used in this study and are listed in Table S1 (Rothrock et al., 2019). Briefly, the farm was about 3 acres in size. The day‐old broilers were transported to the farm, where other poultry and farm animals were also reared, including layer hens, guinea hens, dairy goats and sheep. A small swine herd was also present on an adjacent, but separate plot of land (Rothrock et al., 2019). Fresh fecal samples were collected from the pen area. At the same time, any fecal samples from other animal species surrounding the broiler area on the farm were also collected. Cecal samples were collected after exsanguination. Rinsates were generated by rinsing the carcasses with 100 ml of 10 mM phosphate‐buffered saline in sterile individual bags. All samples were placed on ice at the farm and transported to the laboratory. In the laboratory, isolation and identification of *C. jejuni* were performed according to the standard procedures. Bacterial cultures stored in 15% glycerol at –80°C were revived in Müeller–Hinton agar plates at 42°C for 48 h under microaerobic conditions as described previously (Hiett et al., 2008; Yeh et al., 2013).

Genomic DNA was isolated using a DNeasy Blood and Tissue kit (Qiagen Inc., Germantown, MD, USA) according to the manufacturer's instructions. The quality and quantity of genomic DNA were determined by agarose gel electrophoresis and a spectrophotometer (DS‐11 FX spectrophotometer; DeNovix Inc., Wilmington, DE, USA), respectively. The DNA in 10 mM Tris–HCl (pH 8.0) was stored at –80°C.

### 2.2 PCR amplification of *C. jejuni* CRISPR and sequencing

The PCR primers and conditions for amplification of the *C. jejuni* CRISPRs were described previously (Price et al. 2007). The amplicons were purified with a DNA Clean & Concentrator‐5^™^ kit (Zymo Research, Irvine, CA, USA). The purity of the PCR products was examined with agarose gel electrophoresis. The purified amplicons were sent for DNA sequencing at the USDA ARS Genomics and Bioinformatics Research Unit (Stoneville, MS, USA), where Big Dye terminator chemistry on an ABI 3100 Genetic Analyzer (Thermo Scientific, Foster City, CA, USA) was used. Sequence chromatograms were edited for quality. The same primer pairs for amplification and sequencing were as follows (Price et al. 2007): CRISPR‐F 5′‐GCAACCTCCTTTTAGTGGAGTAATTAG‐3′ and CRISPR‐R 5′‐AAGCGGTTTTAGGGGATTGTAAC‐3′.

### 2.3 Analysis of CRISPR sequences

CRISPR sequences were submitted to the CRISPR Web Server and were identified using the CRISPRFinder program (Grissa et al., 2007; https://crispr.i2bc.paris‐saclay.fr/). Simpson's index of diversity was calculated based on the Hunter and Gaston equation to determine the discriminatory power of genotyping methods (Carriço et al. 2006). The sequences were deposited in GenBank, and the accession numbers are MT199732–MT199808 (Table S1).

## 3 RESULTS AND DISCUSSION

**FIGURE 1:**
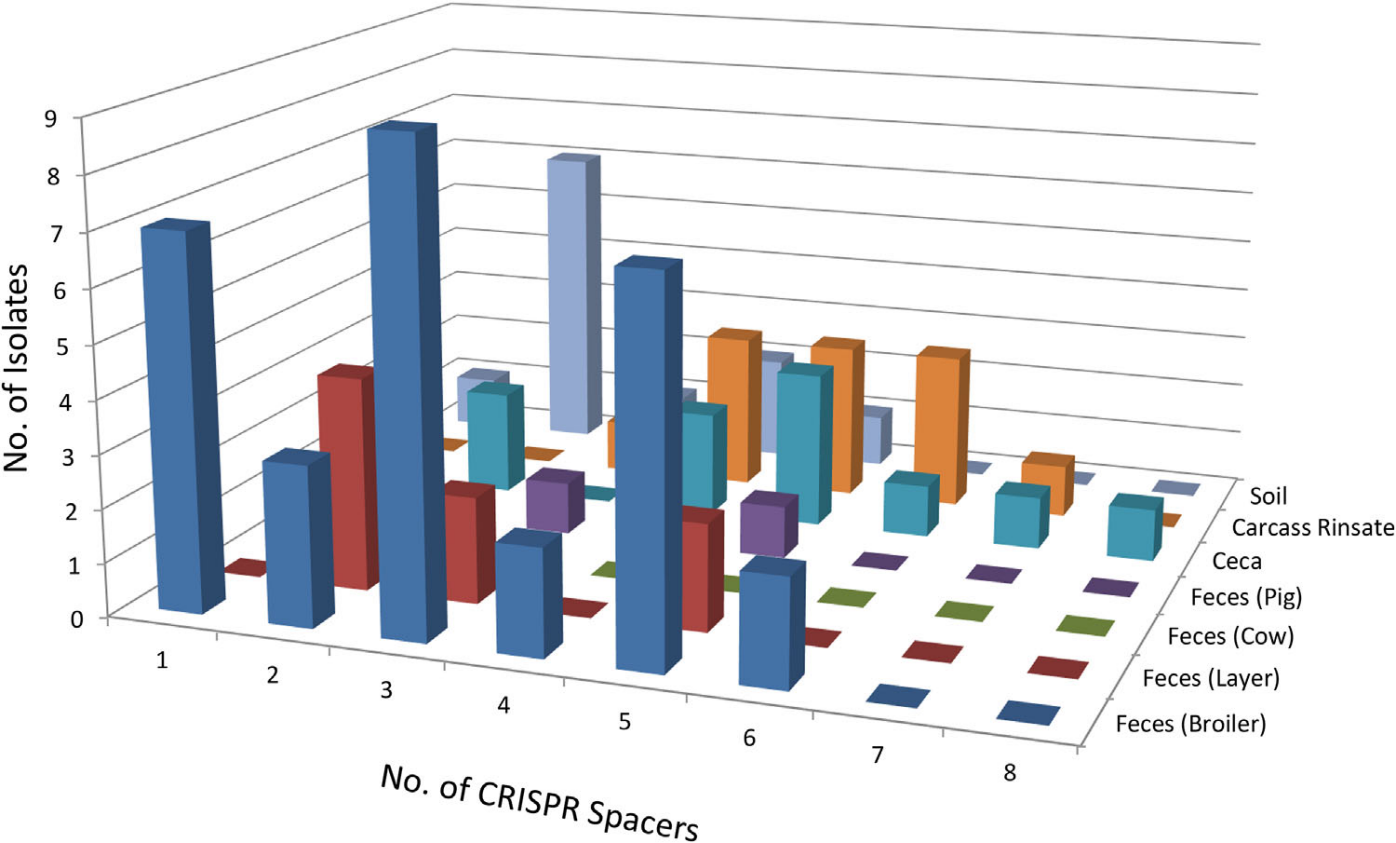
Distribution of the numbers of CRISPR spacers in *Campylobacter jejuni* isolates from a pastured farm in 2016. The *x*‐axis indicates the numbers of CRISPR spacers. The *y*‐axis represents the numbers of *Campylobacter jejuni* isolates

Seventy‐seven PCR amplified products with various sizes were obtained and subjected for sequencing by Big Dye terminator chemistry on an ABI 3100 Genetic Analyzer. The sequences were then submitted to the CRISPRs web server (Grissa et al., 2007) to locate DRs and spacer sequences from the isolates. Grissa et al. (2007) defined the sequences consisting of at least three motifs and at least two exactly identical DRs are regarded as ‘confirmed’ CRISPR, whereas the remaining is considered as ‘questionable’ CRISPR. Our results show that all 77 isolates had either confirmed or questionable CRISPRs with various lengths (Table S1). The DRs varied in lengths, but had a consensus sequence: 5′‐ATTTTACCATAAAGAAATTTAAAAAGGGACTAAAA‐3′. It seems to be common that one end at the DR was not totally conserved (de Cárdenas et al., 2015). A total of 266 spacer sequences were detected in 77 isolates. The numbers of spacers of each isolate range from one spacer to eight spacers (Table S1). By analysing these spacer sequences with the MUSCLE alignment program (Edgar, 2004), we observed 67 different spacer sequences in 77 *C. jejuni* isolates (Table 1). Further comparing the current space sequences with those from previous reports from poultry sources, such as cecal content and neck skin of broilers, laying hens from organic farms and sewage water (de Cárdenas et al., 2015; Kovanen et al., 2014; Louwen et al., 2013), shows that 18 out of 67 space sequences were identified previously, whereas 49 sequences were unique to isolates from this study. Among 67 space sequences, four sequences (Nos. 16, 19, 48 and 57) were found in the same 15 isolates (Table 1). The sequence Nos. 16, 19 and 48 (Table 1) were identified in the previous report (de Cárdenas et al., 2015), but No. 57 sequence was first identified in this study. The distributions of the numbers of CRISPR spacers from different sources seemed to be random. Overall, 17 out of 77 (22%) *C. jejuni* isolates had two and five spacers, whereas 14 out of 77 (18%) isolates had three spaces in their genomes (Figure 1). Because the spacer sequences are often the indicators of the phage invasion, high polymorphic nature of the CRISPR sequences in the *C. jejuni* genomes is expected.

**TABLE 1:** Spacer alleles of *Campylobacter jejuni* isolates in this study

No.	Spacer sequence (5′‐ > 3′)	Isolates	Reference
1	TACATTTACTTAAGTCTTTAAACTCAGGGT	K2‐34	This study
2	TATAGAATGGAGCATTTAGAAGAAGATAT	I10‐31A, I10‐33A	This study
3	(CT)GAGTTACCAAGATTAAAACTTCCTATGA	B5‐13, J1‐13A, J1‐16, J2‐1A, J2‐13C, J2‐5A, JE2‐2A, JE2‐4A	de Cárdenas et al. (2015)
4	CAATAATGGAGAACATTTTGATAGAGGCAGGAT	E5‐36	This study
5	ATAATGGCTAAATATTTCATGAGAATGGA	E5‐22A, E5‐23A, E5‐35, E5‐38, E5‐42A, E5‐44A, J2‐36	de Cárdenas et al. (2015)
6	CAATAATGGCTAAATATTTCATGAGAATGGA	K1‐4A, K2‐15, K2‐22, K2‐23	This study
7	ACTAAAGCACCATTGTATTTTACAATTAAA	I10‐31A, I10‐33A	This study
8	TAGTAGCTAAGAATAAAATAAGAAACACTGG	J2‐45A	This study
9	TTAGAGTATAGAGTAAATAAGAAAGAAAC	E5‐22A, E5‐23A, E5‐35, E5‐38, E5‐42A, E5‐44A, J2‐36	This study
10	CACCAGGAGTTTGAGGAAATAAGAAAGAGTC	E5‐36	This study
11	TCTATATCAGAATATGTCGAAAATGAATTA	B5‐12, B5‐17, B5‐19, B5‐22A, B5‐34, B5‐39, J2‐40	This study
12	TCTTAATCTCTTCACATTTTCTTTTGAGTAT	I10‐1A	This study
13	TAAAAAGTATTATAAGTTCAGCGTTTAATT	L2‐12	This study
14	CAGCTACTAATGAAAATGAAACAATTTTAGA	L1‐4A, L1‐5, L1‐7	This study
15	ATTTTATTCTTAGCCACTATTTCAATCTT	E5‐22A, E5‐23A, E5‐35, E5‐38, E5‐42A, E5‐44A, J2‐36	de Cárdenas et al. (2015)
16	CCAGTGTTTCTTATTTTATTCTTAGCTACTA	EP2‐3A, J1‐12, J1‐22A, J1‐25A, J1‐32, J2‐22A, J2‐24A, J2‐32, J2‐35, J2‐44A, JE1‐2A, JE1‐9, K1‐21H, K2‐39, KE1‐2A	de Cárdenas et al. (2015)
17	ATTAATCCATATAAATTCCCTACCATCAA	E5‐22A, E5‐23A, E5‐35, E5‐38, E5‐42A, E5‐44A, J2‐36	This study
18	CTATAAATCACCACATGTAGAGAGTGAATCA	E5‐36	This study
19	CTGAAAGGTTATAAATGAAATTAGAAATTAT	EP2‐3A, J1‐12, J1‐22A, J1‐25A, J1‐32, J2‐22A, J2‐24A, J2‐32, J2‐35, J2‐44A, JE1‐2A, JE1‐9, K1‐21H, K2‐39, KE1‐2A	de Cárdenas et al. (2015)
20	TACTGAAGTAAAATAAGTAGTAGAAATTAC	I10‐31A, I10‐33A	This study
21	ACTTATTGCAACTGAAGTAAAAGGAATCGG	I10‐31A, I10‐33A	This study
22	CTGAAATAACTTCTAAATTCTAATACAATAT	K1‐4A, K2‐15, K2‐22, K2‐23	This study
23	CCTATTTGATAATCTTTGAAAATTCTAA	I10‐33A	This study
24	CGTCAACCTCTAAGCTTTGCGCCATATTGG	I10‐15A	This study
25	CAAATCAACTTCTAAGCTATCATCAAATTT	I10‐31A, I10‐33A	This study
26	CTTCTTTTGTCTCATAACCCACTCAACAAAA	E5‐32, K2‐3A, K2‐19C, K2‐19E, K2‐33, K2‐37	de Cárdenas et al. (2015)
27	CTTACTACACAGCCAGTCGTGTATAACGCA	K1‐4A, K2‐15, K2‐22, K2‐23	This study
28	AACCCTAGTGGATTGAAACTCCGCTAGGGCTAATTACTCCACTAAAGGAAGGTTTGCACAAACTAATGTGAAATTGAACTCCGCAAGGGAT	E5‐44A	This study
29	ACCCTAGTGGATTGAAACTCCGCTAGGGCTAATTACTCCACTAAAGGAGGTTGCAAAATACCCTAACACCTCTTAAATCATCGAGCTGCTA	K2‐37	This study
30	ATAAGAGACCACATTTATAGCGTTAAACA	E5‐22A, E5‐23A, E5‐35, E5‐38, E5‐42A, E5‐44A, J2‐36	This study
31	ATAATTTCTAATTTCATTTATAACCTTTCAG	J2‐45A	This study
32	(CA)TGAGAACTTAAATAAGTTTATCAAAGATA	B5‐13, J1‐13A, J1‐16, J2‐1A, J2‐13C, J2‐5A, JE2‐2A, JE2‐4A	de Cárdenas et al. (2015)
33	CATTTGCGTTTGCATTATTAATAACGCTACT	K2‐2, K2‐3, KB1‐4A, KB1‐5A, KB1‐7A, KB1‐10A, KE1‐5A, KE2‐5A, KE2‐6A, KE2‐6D	de Cárdenas et al. (2015)
34	GAAACCCAGATTAAATGATCGTTTGAGA	I10‐15A	This study
35	CTTTACAATATTGTAAAAACATAAAAGTGG	L2‐12	de Cárdenas et al. (2015)
36	CTACTTGATTATCATTATACTCTAAAGGTTC	B5‐24A, B5‐31	de Cárdenas et al. (2015)
37	CTTCTGATGTTATAATTACATTAGATAAATC	JE1‐1	This study
38	(CT)AATGCTTTGATTATAAAAATTACATAAA	B5‐13, J1‐13A, J1‐16, J2‐1A, J2‐13C, J2‐5A, JE2‐2A, JE2‐4A	de Cárdenas et al. (2015)
39	CTTATACTTTGATTATAAAAATTACATAAAG	K2‐2, K2‐3, KB1‐4A, KB1‐5A, KB1‐7A, KB1‐10A, KE1‐5A, KE2‐5A, KE2‐6A, KE2‐6D	This study
40	CTTATTTATGCGGTGCAAGTCAAGTTGAAAC	E5‐32, K2‐3A, K2‐19C, K2‐19E, K2‐33	This study
41	ATTTATGCGGTGCAAGTCAAGTTGAAAC	K2‐37	This study
42	TGGTTATTTATTTGGGGCTGATATTGGTTC	I10‐31A	This study
43	TGGTTATTTATTTGGTGCTGATATTGGTTC	I10‐33A	This study
44	TCTAAAGCGCTTGCTATTGAAGTTTTATTG	I10‐12A, I10‐23A, I10‐24A, IP2‐1A, IP2‐2A	This study
45	CTCGTGCTATTGTTTTAGCTCGACGATTT	E5‐23A, E5‐35, E5‐38, E5‐42A, E5‐44A, J2‐36, E5‐22A	Louwen et al. (2010)
46	TAATTCATTTTCGACATATTCTGATATAGA	K2‐34	This study
47	GTTGGAATGCTTAAGCAGGGGTGGAGTGAAG	J2‐45A	This study
48	CTTCACTCCACCCCTGCTTAAGCATTCCAAC	EP2‐3A, J1‐12, J1‐22A, J1‐25A, J1‐32, J2‐22A, J2‐24A, J2‐32, J2‐35, J2‐44A, JE1‐2A, JE1‐9, K1‐21H, K2‐39, KE1‐2A	de Cárdenas et al. (2015)
49	CCTGCTAAAGAACATACTGTTAAAGCATCT	B5‐12, B5‐17, B5‐19, B5‐22A, B5‐34, B5‐39, J2‐40	de Cárdenas et al. (2015)
50	TTGCTTCGTTCAATCAAAAACAGGTGCA	I10‐15A	This study
51	CTTCCCAATCGCAAAGCAATAATCCTTTTAAC	J2‐22A, JE1‐2A, JE1‐9	de Cárdenas et al. (2015)
52	CTTCCCAATCGCAAAGCAAAATCCTTTTAAC	EP2‐3A, J1‐12, J1‐22A, J1‐25A, J1‐32, J2‐24A, J2‐32, J2‐35, J2‐44A, K1‐21H, K2‐39, KE1‐2A	Louwen et al. (2010)
53	CTAGCGAAAACAATTTAAATAAAGCAAAATT	JE1‐1	This study
54	GTTAAAAGGATTTTGCTTTGCGATTGGGAAG	J2‐45A	This study
55	TGTATAAGCTTGTGCTGTAGTAATTTCAAT	EP2‐5A	Louwen et al. (2010)
56	CCAGTGTATTAAAATTGCACGACTTGCTGG	EP2‐5A	This study
57	CTCTTAATTTCAACAATACTCACTTATTAAAT	JE1‐9, EP2‐3A, J1‐22A, J1‐25A, J1‐32, J2‐22A, J2‐24A, J2‐32, J2‐35, J2‐44A, JE1‐2A, K1‐21H, K2‐39, KE1‐2A, J1‐12	This study
58	TATAATACCATTCTTAATTTAAAAGGAGTG	B5‐12, B5‐17, B5‐19, B5‐22A, B5‐34, B5‐39, J2‐40	de Cárdenas et al. (2015)
59	TTATAATACCATTAGCCATTAAAACGGAGTG	E5‐36	This study
60	AGATGCTTTAACAGTATGTTCTTTAGCAGG	K2‐34	This study
61	TTTCCATTTGCATCCACCTCCAATTTTTCTA	I10‐31A, I10‐33A	This study
62	AAGCTTGTACTTAAATGACATTCATAAA	I10‐15A	This study
63	AAGCTTGCCCTTAAATGACAATCATAAA	I10‐15A	This study
64	GTCATTTTTAATCCTTAAGTAAGTAATAAT	EP2‐5A	This study
65	CACTCCTTTTAAATTAAGAATGGTATTATA	K2‐34	This study
66	ACCCTGAGTTTAAAGACTTAAGTAAATGTA	B5‐12, B5‐17, B5‐19, B5‐22A, B5‐34, B5‐39, J2‐40	This study
67	ATTTAATAAGTGGTATTGTTGAAATTAAGAG	J2‐45A	This study

The high‐resolution melting analysis (HRMA) and DNA sequencing have been used in CRISPR studies (Gomes et al., 2016; Price et al., 2007). Because the spacer sequences are often parts of the sequences from plasmids or phages, the DNA sequencing provides more accurate information for building the database for comparison from different laboratories. Gomes et al. (2016) compared the genotyping methods and found the discriminative indices were 0.916 and 0.550, respectively, for the DNA sequencing and HRMA.

The multi‐locus sequence typing (MLST) (Dingle et al., 2001) has been the most used for genotyping foodborne bacterial pathogens. In our unpublished observations, we found 13 MLST sequence types (ST) in 66 isolates (Table S1). Among them, ST‐607 and ST‐353 were detected in 17 and 16 isolates, respectively, in this study. Further analysis shows ST‐607 had five CRISPR spacer sequence patterns: six isolates contained Nos. 11, 49, 58 and 66 spacer sequences, seven isolates contained Nos. 3, 32 and 38 sequences, two isolates contained No. 44 sequence, one isolate contained Nos. 37 and 53 sequences and one isolate contained Nos. 16, 19, 48, 52 and 57 sequences (Table S1). It is also observed that ST‐353 had four CRISPR space sequence patterns: six isolates had Nos. 5, 9, 15, 17, 30 and 45 spacer sequences, eight isolates had Nos. 33 and 39 sequences, one isolate had Nos. 4, 10, 18 and 59 sequences and one isolate had Nos. 26 and 40 sequences. Further, the CRISPR types of our current isolates were assigned based on the spacer numbers and sequences, and Simpson's index of diversity was calculated to measure the discriminatory power of these methods. As shown in Table 2, a combination of both methods had the Simpson's index value of 0.953 that is higher than those of 0.922 or 0.849 for CRISPR or MLST, respectively. These results suggest that the MLST can further be discriminated based on CRISPR spacer sequences and the numbers of spacers. Further investigation on the CRISPR molecular variations in and the numbers of spacers to increase the resolution is needed.

**TABLE 2:** Simpson's index diversity of genotyping methods

Method	Number of genotypes	Simpson's index of diversity^a^	95% Confidence interval^a^
MLST	13	0.849	0.809 ‐ 0.889
CRISPR	21	0.922	0.899 ‐ 0.945
CRISPR + MLST	32	0.953	0.934 ‐ 0.971

^a^ Both were calculated using the online tool based on the Hunter and Gaston equation.

In summary, *C. jejuni* isolates from 2016 and an animal farm were subjected to CRISPR type 1 analysis. The CRISPR sequences were identified in all 77 isolates. One type of DR was detected in the CRISPR sequences. The lengths of the CRISPR sequences ranged from 100 to 560 nucleotides. The number of spacers ranged from one to eight. By further analysis of spacer sequences, a total of 266 sequences were identified from 77 *C. jejuni* isolates. Among them, 67 distinctive sequences were identified. Furthermore, by comparison with known spacer sequences, we observed that 18 from 67 sequences were known previously and 49 sequences were unique in this study. Further analysis shows that the MLST from *C. jejuni* isolates can be discriminated based on CRISPR spacer sequences and the numbers of spacers. In the future, investigation on the CRISPR resolution for *C. jejuni* identification in outbreaks is needed. A database that integrates both MLST sequences and CRISPR sequences and is searchable is greatly in demand. During the revision of this manuscript, a study demonstrates the CRISPR–Cas system is prevalent in the fluoroquinolone‐resistant *C. jejuni* isolates (Adiguzel et al., 2021).

## AUTHOR CONTRIBUTIONS

Hung‐Yueh Yeh: Conceptualization, Data curation, Formal analysis, Investigation, Methodology, Supervision, Writing‐original draft, Writing‐review & editing. Amal Awad: Formal analysis, Investigation, Methodology. Michael Rothrock: Resources

## CONFLICT OF INTEREST

The authors declare no conflict of interest.

### PEER REVIEW

The peer review history for this article is available at https://publons.com/publon/10.1002/vms3.622

## Supporting information

Supporting Information

## Data Availability

The sequences were deposited in GenBank, and the accession numbers are MT199732–MT199808.

## References

[vms3622-bib-0001] Adiguzel, M. C. , Goulart, D. B. , Wu, Z. , Pang, J. , Cengiz, S. , Zhang, Q. , & Sahin, O. (2021). Distribution of CRISPR types in fluoroquinolone‐resistant Campylobacter jejuni isolates. Pathogens 10, 345. 10.3390/pathogens10030345 33809410 PMC8000906

[vms3622-bib-0002] Ahmed, W. , Hafeez, M. A. , Ahmad, R. , & Mahmood, S. (2018). CRISPR‐Cas system in regulation of immunity and virulence of bacterial pathogens. Gene Rep 13, 151–157. 10.1016/j.genrep.2018.10.004

[vms3622-bib-0003] Barrangou, R. , & Horvath, P. (2009). The CRISPR system protects microbes against phages, plasmids. Microbe 4, 224–230. 10.1128/microbe.4.224.1

[vms3622-bib-0004] Carriço, J. A. , Silva‐Costa, C. , Melo‐Cristino, J. , Pinto, F. R. , de Lencastre, H. , Almeida, J. S. , & Ramirez, M. (2006). Illustration of a common framework for relating multiple typing methods by application to macrolide‐resistant *Streptococcus pyogenes* . Journal of Clinical Microbiology 44, 2524–2532. 10.1128/JCM.02536-05 16825375 PMC1489512

[vms3622-bib-0005] Cox, N. A. , Berrang, M. E. , House, S. L. , Medina, D. , Cook, K. L. , & Shariat, N. W. (2019). Population analyses reveal preenrichment method and selective enrichment media affect *Salmonella* serovars detected on broiler carcasses. Journal of Food Protection 82, 1688–1696. 10.4315/0362-028X.JFP-19-166 31536420

[vms3622-bib-0006] Crim, S. M. , Griffin, P. M. , Tauxe, R. , Marder, E. P. , Gilliss, D. , Cronquist, A. B. , Cartter, M. , Tobin‐D'Angelo, M. , Blythe, D. , Smith, K. , Lathrop, S. , Zansky, S. , Cieslak, P. R. , Dunn, J. , Holt, K. G. , Wolpert, B. , & Henao, O. L. (2015). Preliminary incidence and trends of infection with pathogens transmitted commonly through food ‐ foodborne diseases active surveillance network, 10 U.S. sites, 2006–2014. Morb Mortal Wkly Rep 64, 495–499. PMC4584825 25974634

[vms3622-bib-0007] de Cárdenas, I. , Fernández‐Garayzábal, J. F. , de la Cruz, M. ‐. L. , Domínguez, L. , Ugarte‐Ruiz, M. , & Gómez‐Barrero, S. (2015). Efficacy of a typing scheme for *Campylobacter* based on the combination of true and questionable CRISPR. Journal of Microbiological Methods 119, 147–153. 10.1016/j.mimet.2015.10.020 26518609

[vms3622-bib-0008] Dingle, K. E. , Colles, F. M. , Wareing, D. R. , Ure, R. , Fox, A. J. , Bolton, F. E. , Bootsma, H. J. , Willems, R. J. , Urwin, R. , & Maiden, M. C. (2001). Multilocus sequencing typing system for *Campylobacter jejuni* . Journal of Clinical Microbiology 39, 14–23. 10.1128/JCM.39.1.14-23.2001 11136741 PMC87672

[vms3622-bib-0009] Edgar, R. C. (2004). MUSCLE: multiple sequence alignment with high accuracy and high throughput. Nucleic Acids Research 32, 1792–1797. 10.1093/nar/gkh340 15034147 PMC390337

[vms3622-bib-0010] European Food Safety Authority (2010). The community summary report on trends and sources of zoonoses, zoonotic agents and food‐borne outbreaks in the European Union in 2008. EFSA Journal 8, 1496. 10.2903/j.efsa.2010.1496 PMC13129928 42077618

[vms3622-bib-0034] Gomes, C. N. , Souza, R. A. , Passaglia, J. , Duque, S. S. , Medeiros, M. I. C. , & Falcao, J. P. (2016). Genotyping of *Campylobacter coli* strains isolated in Brazil suggests possible contamination amongst environmental, human, animal and food sources. Journal of Medical Microbiology 65, 80–90. 10.1099/jmm.0.000201 26531157

[vms3622-bib-0011] Grissa, I. , Vergnaud, G. , & Pourcel, C. (2007). CRISPRFinder: a web tool to identify clustered regularly interspaced short palindromic repeats. Nucleic Acids Research 35, W52‐57. 10.1093/nar/gkm360 17537822 PMC1933234

[vms3622-bib-0012] Grissa, I. , Vergnaud, G. , & Pourcel, C. (2009). Clustered regularly interspaced short palindromic repeats (CRISPRs) for the genotyping of bacterial pathogens. Methods in Molecular Biology 551, 105–116. 10.1007/978-1-60327-999-4_9 19521870

[vms3622-bib-0013] Hermans, D. , Pasmans, F. , Messens, W. , Martel, A. , Van Immerseel, F. , Rasschaert, G. , Heyndrickx, M. , Van Deun, K. , & Haesebrouck, F. (2012). Poultry as a host for the zoonotic pathogen *Campylobacter jejuni* . Vector Borne and Zoonotic Diseases (Larchmont, N.Y.) 12, 89–98. 10.1089/vbz.2011.0676 22133236

[vms3622-bib-0014] Hiett, K. L. , Stintzi, A. , Andacht, T. M. , Kuntz, R. L. , & Seal, B. S. (2008). Genomic differences between Campylobacter jejuni isolates identify surface membrane and flagellar function gene products potentially important for colonizing the chicken intestine. Functional & Integrative Genomics 8, 407–420. 10.1007/s10142-008-0087-6 18592283

[vms3622-bib-0015] Hille, F. , Richter, H. , Wong, S. P. , Bratovič, M. , Ressel, S. , & Charpentier, E. (2018). The biology of CRIPR‐Cas: background and forward. Cell 171, 1239–1259. 10.1016/j.cell.2017.11.032 29522745

[vms3622-bib-0016] Ishino, Y. , Krupovic, M. , & Forterre, P. (2018). History of CRISPR‐Cas from encounter with a mysterious repeated sequence to genome editing technology. Journal of Bacteriology 200, e00580‐17. 10.1128/JB.00580-17 29358495 PMC5847661

[vms3622-bib-0017] Ishino, Y. , Shinagawa, H. , Makino, K. , Amemura, M. , & Nakata, A. (1987). Nucleotide sequence of the *iap* gene, responsible for alkaline phosphatase isozyme conversion in *Escherichia coli*, and identification of the gene product. Journal of Bacteriology 169, 5429–5433. 10.1128/jb.169.12.5429-5433.1987 3316184 PMC213968

[vms3622-bib-0018] Kirk, M. D. , Pires, S. M. , Black, R. E. , Caipo, M. , Crump, J. A. , Devleesschauwer, B. , Döpfer, D. , Fazil, A. , Fischer‐Walker, C. L. , Hald, T. , Hall, A. J. , Keddy, K. H. , Lake, R. J. , Lanata, C. F. , Torgerson, P. R. , Havelaar, A. H. , & Angulo, F. J. (2015). World Health Organization estimates of the global and regional disease burden of 22 foodborne bacterial, protozoal, and viral diseases, 2010: A data synthesis. Plos Medicine 12, e1001921. 10.1371/journal.pmed.1001921 26633831 PMC4668831

[vms3622-bib-0019] Kovanen, S. M. , Kivistö, R. I. , Rossi, M. , & Hänninen, M. ‐. L. (2014). A combination of MLST and CRISPR typing reveals dominant *Campylobacter jejuni* types in organically farmed laying hens. Journal of Applied Microbiology 117, 249–257. 10.1111/jam.12503 24655229

[vms3622-bib-0020] Lin, J. (2009). Novel approaches for *Campylobacter* control in poultry. Foodborn Pathog Dis 6, 755–765. 10.1089/fpd.2008.0247 PMC3145176 19425824

[vms3622-bib-0021] Louwen, R. , Horst‐Kreft, D. , de Boer, A. G. , van der Graaf, L. , de Knegt, G. , Hamersma, M. , Heikema, A. P. , Timms, A. R. , Jacobs, B. C. , Wagenaar, J. A. , Endtz, H. P. , van der Oost, J. , Wells, J. M. , Nieuwenhuis, E. E. S. , van Vliet, A. H. M. , Willemsen, P. T. J. , van Baarlen, P. , & van Belkum, A. (2013). A novel link between *Campylobacter jejuni* bacteriophage defence, virulence and Guillain‐Barré syndrome. European Journal of Clinical Microbiology & Infectious Diseases 32, 207–226. 10.1007/s10096-012-1733-4 22945471

[vms3622-bib-0022] Louwen, R. , Staals, R. H. J. , Endtz, H. P. , van Baarlen, P. , & van der Oost, J. (2014). The role of CRISPR‐Cas systems in virulence of pathogenic bacteria. Microbiology and Molecular Biology Reviews 78, 74–88. 10.1128/MMBR.00039-13 24600041 PMC3957734

[vms3622-bib-0023] On, S. L. W. , & Jordan, P. J. (2003). Evaluation of 11 PCR assays for species‐level identification of *Campylobacter jejuni* and *Campylobacter coli* . Journal of Clinical Microbiology 41, 330–336. 10.1128/jcm.41.1.330-336.2003 12517869 PMC149560

[vms3622-bib-0024] Price, E. P. , Smith, H. , Huygens, F. , & Giffard, P. M. (2007). High‐resolution DNA melt curve analysis of the clustered, regularly interspaced short‐palindromic‐repeat locus of *Campylobacter jejuni* . Applied and Environmental Microbiology 73, 3431–3436. 10.1128/AEM.02702-06 17400785 PMC1907115

[vms3622-bib-0025] Rothrock, M. J. , Locatelli, A. , Feye, K. M. , Caudill, A. J. , Guard, J. , Hiett, K. , & Ricke, S. C. (2019). A microbiomic analysis of a pasture‐raised broiler flock elucidates foodborne pathogen ecology along the farm‐to‐fork continuum. Front Vet Sci 6, 260. 10.3389/fvets.2019.00260 31448296 PMC6692657

[vms3622-bib-0026] Ryan, K. J. , Ray, C. G. , & Sherris, J. C. (2004). Sherris medical microbiology: an introduction to infectious diseases, 4th edn. McGraw‐Hill, New York

[vms3622-bib-0027] Sahin, O. , Kassem, I. I. , Shen, Z. , Lin, J. , Rajashekara, G. , & Zhang, Q. (2015). *Campylobacter* in poultry: ecology and potential interventions. Avian Diseases 59, 185–200. 10.1637/11072-032315-Review 26473668

[vms3622-bib-0028] Scallan, E. , Hoekstra, R. M. , Angulo, F. J. , Tauxe, R. V. , Widdowson, M. A. , Roy, S. L. , Jones, J. L. , & Griffin, P. M. (2011). Food‐borne illness acquired in the United States – major pathogens. Emerging Infectious Diseases 17, 7–15. 10.3201/eid1701.P11101 21192848 PMC3375761

[vms3622-bib-0029] Shariat, N. , & Dudley, E. G. (2014). CRISPRs: molecular signatures used for pathogen subtyping. Applied and Environmental Microbiology 80, 430–439. 10.1128/AEM.02790-13 24162568 PMC3911090

[vms3622-bib-0030] Tack, D. M. , Marder, E. P. , Griffin, P. M. , Cieslak, P. R. , Dunn, J. , Hurd, S. , Scallan, E. , Lathrop, S. , Muse, A. , Ryan, P. , Smith, K. , Tobin‐D'Angelo, M. , Vugia, D. J. , Holt, K. G. , Wolpert, B. J. , Tauxe, R. , & Geissler, A. L. (2019). Preliminary incidence and trends of infections with pathogens transmitted commonly through food—foodborne diseases active surveillance network, 10 U.S. sites, 2015–2018. Morb Mortal Wkly Rep 68, 369–373. 10.15585/mmwr.mm6816a2 PMC6483286 31022166

[vms3622-bib-0031] Ursing, J. B. , Lior, H. , & Owen, R. J. (1994). Proposal of minimal standards for describing new species of the family *Campylobacteraceae* . International Journal of Systematic Bacteriology 44, 842–845. 10.1099/00207713-44-4-842 7981111

[vms3622-bib-0032] Yeh, H. , Hiett, K. L. , Line, J. E. , Oakley, B. B. , & Seal, B. S. (2013). Construction, expression, purification and antigenicity of recombinant *Campylobacter jejuni* flagellar proteins. Microbiological Research 168, 192–198. 10.1016/j.micres.2012.11.010 23312848

